# Dual Chamber Pacing in a Patient of Hypertrophic Cardiomyopathy with Failure to Wean from Mechanical Ventilator

**Published:** 2008-11-01

**Authors:** Shaffi Kanjwal, Khalil Kanjwal, Naser Imran, Yousuf Kanjwal

**Affiliations:** University of Toledo Medical Center

**Keywords:** hypertrophic cardiomyopathy, pulmonary edema, dual chamber pacemaker

## Abstract

We discuss the case of a 63 years old female who required repeated intubation due to recurrent pulmonary edema. She was found to have hypertrophic cardiomyopathy with a gradient of 82 mmHg across the left ventricular outflow tract. Initially adequate rate control and treatment with negative inotropes did not help her condition. Finally a dual chamber pacemaker implantation and atrioventricular node modification lead to successful extubation.

## Background

Hypertrophic cardiomyopathy has been reported as a cause of failure to wean from mechanical ventilator [1].We report a case of hypertrophic cardiomyopathy with recurrent failures to wean from a mechanical ventilator. Patient underwent dual chamber pacemaker implantation and AV node modification, with improvement in left ventricular outflow tract (LVOT) gradient. Patient was subsequently successfully extubated.

## Case

**Figure 1:**
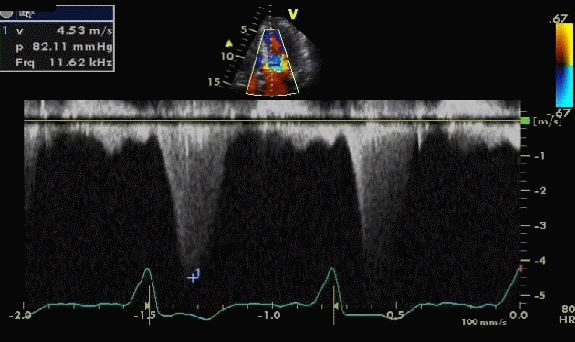
Continuous wave (CW) Doppler across the left ventricular outflow tract showing a gradient of 82.2 mmHg prior to insertion of pacemaker.

**Figure 2:**
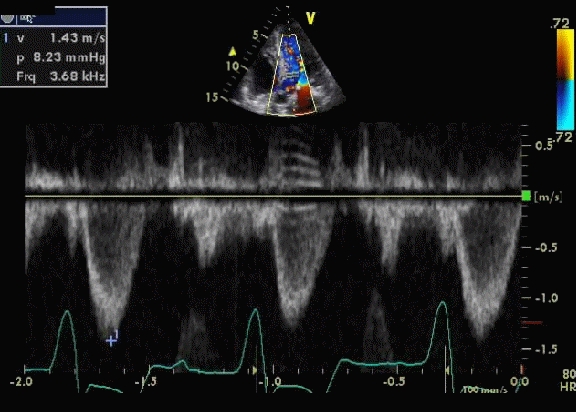
Continuous wave (CW) Doppler image across left ventricular outflow tract during pacing showing a decrease in gradient to 8.2 mmHg ( a decrease of 74 mmHg).

63-year-old Caucasian female, with past medical history of failure of transplanted kidney, persistent atrial fibrillation, hypertension and hypothyroidism was admitted to the intensive care unit with acute respiratory failure secondary to pulmonary edema. She was intubated and treated with intravenous diuretics. An echocardiogram revealed severe hypertrophic cardiomyopathy with a left ventricular outflow gradient of 82 mm Hg (Figure 1). She went through a protracted course of critical illness and multiple trials of extubation had failed. No other cause like electrolyte disturbance, sepsis or any other metabolic cause could be determined. She received multiple medications for rate control in addition to negative ionotrope like disopyramide. Multiple cardio versions were attempted without any success. Patient continued to need reintubation despite adequate rate control and at that time a decision was made to perform atrioventricular node ablation and a pacemaker implant. The outflow gradient decreased dramatically following the procedure from 82 mmHg to 8 mmHg within next few days and patient showed significant improvement and was successfully extubated and transferred out of the intensive care unit (Figure 2).

## Discussion

Lung congestion and low cardiac output syndromes are commonly encountered in critically ill and mechanically ventilated patients. These disturbances may impede weaning from mechanical ventilator and require repeated intubations if left unaddressed. Proper management of congestive heart failure in mechanically ventilated patients has been associated with favorable outcomes, and by optimizing the treatment patients have successfully been weaned from mechanical ventilators. Hypertrophic Cardiomyopathy (HOCM) may be one of the conditions responsible for such a scenario and the routine treatment for systolic heart failure may be detrimental in such patients.

Adamopoulos et al [1] reports a series of two patients with congestive heart failure and difficulty weaning from mechanical ventilator. Both these patients were subsequently found to have hypertrophic cardiomyopathy on echocardiography and following treatment with negative inotropic agents, successful weaning was possible.

In patients who are refractory to medical management permanent pacing in the form of dual chamber pacing (DDD) has been introduced as an alternative treatment modality. By altering the pattern of ventricular depolarization pacing may result in decreased left ventricular outflow tract gradient and improve functional status in such patients [2].The improvement in symptoms and hemodynamic indices occur during the first few weeks to months of dual chamber pacing and favorable changes are often observed upto 3 years. In an another series of 84 patients, Fananapazir etal [3] reports significant improvement in functional class and mortality in patients of hypertrophic cardiomyopathy following  implantation of dual chamber pacemaker. There was a significant improvement in both resting as well as provokaable LVOT gradients.

Our patient in addition to hypertrophic cardiomyopathy had atrial fibrillation which might have independently contributed to worsening of the outflow gradient. Inspite of good rate control during atrial fibrillation in addition to negative inotropic medication, patient continued to have repeated intubations. Although alcohol septal ablation is a recommended procedure in a selected group of patients, it was not contemplated in this patient who was critically sick. The long-term results of dual chamber pacing in hypertrophic cardiomyopathy are controversial [3,4]. There are some reports of regression of hypertrophy with dual chamber pacing, there by improving symptoms. Our aim was to achieve acute hemodynamic benefit in this patient. We believe it was the combination of definite rate control and improvement in outflow gradient that finally lead to successful extubation. This observation is consistent with other studies [3].

## Conclusion

In patients with difficult weaning and extubation from a mechanical ventilator due to congestive heart failure in an intensive care unit setting, an underlying cause including hypertrophic cardiomyopathy should be ruled out.
